# Supplementary material for: A new framework for facial age estimation in humans and AIs
Source: Sci Rep. 2026 May 11;16:21439. doi: 10.1038/s41598-026-49573-1 (PMC13350816; doi:10.1038/s41598-026-49573-1)

Supplementary materials 1: ANOVA subject-based analysis of the results of Experiment 1b

The ANOVA subject-based analysis was performed in a similar manner to the ANOVA item-based analysis in Experiment 1b. Unlike in the item-based analysis, we have now computed for each of the participants the average MAEs and the average MAE-Us for each facial age group. As in the item-based ANOVA, facial ages were grouped into groups of 7 decades (e.g., 18-29, 30-39, 40-49 etc.). For each participant, we first computed MAE (the absolute value of estimated-chronological age) and MAE-U (the absolute value of estimated-average perceived age) for each item and then computed the average MAE-Us and the average MAEs in each of the seven age groups.

A repeated measure ANOVA analysis was used to compare the pattern of results, with type of measure (MAE-Us vs MAEs) and with the specific age group as the two within-subject independent variables. A main effect of measure type (MAE-U vs MAE) [F(1,69) = 342.9, p < 0.001], indicated that the magnitude of error was smaller in MAE-Us compared to MAEs. A main effect for specific age group [F(6,414) = 64.44, p < 0.001], indicated general differences in absolute errors between different age groups. More importantly and as for the item-based analysis, a significant interaction was found between age group and type of measure [F(6,414) = 31.25, p < 0.001]. This finding reinforces the idea that the pattern of errors throughout the lifespan is different between MAE-Us and MAEs. As can be seen in Figure s1, MAEs show a general increase with age throughout the lifespan. In contrast, MAE-Us increase with age up to mid-adulthood and then begin to decrease. As in the item-based ANOVA analysis, we performed specific comparisons between the age group of 70-79 and the age group of 60-69 to test if MAE-Us and MAEs show such a decrease. The results showed that MAE-Us were significantly smaller for the age group of 70-79 [t=2.1, p < 0.05]. The difference between the MAEs between the 70-79 and the 60-69 age groups was not significant [t<1, p > 0.05].

Figure s1: Average MAE-Us and MAEs averaged across different age groups (subject-based analysis)


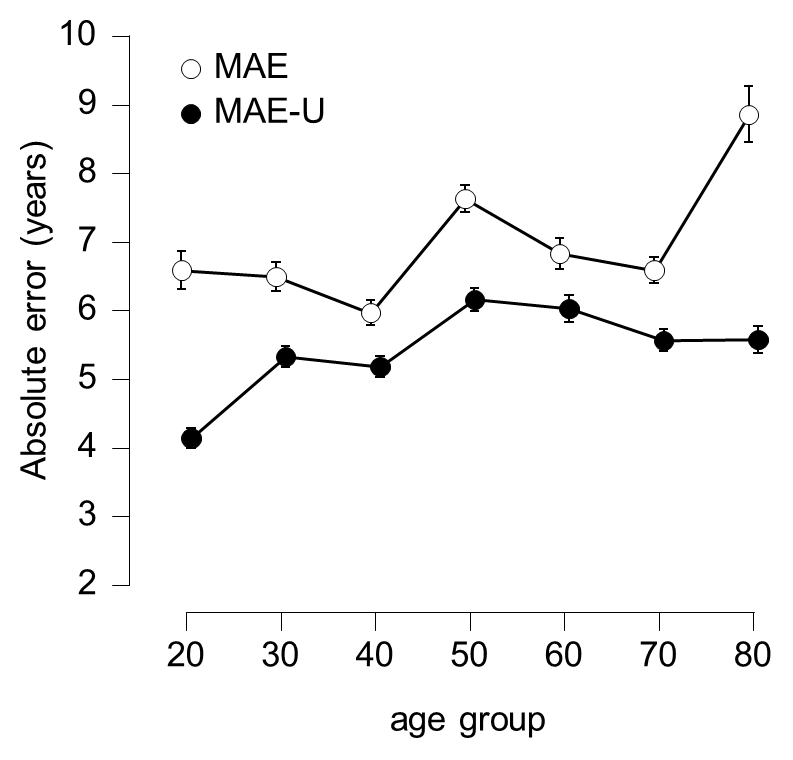

Supplement: Supplementary file 1 — Supplementary Material 1 [file 41598_2026_49573_MOESM1_ESM.docx]
